# Cognitive and behavioral response of mosquitofish (*Gambusia affinis*) to environmental factors: Microplastics, predator cues, and detour design methods

**DOI:** 10.1111/jfb.15998

**Published:** 2024-11-13

**Authors:** Kyndal Irwin, Grace Hathorn, Caitlin R. Gabor

**Affiliations:** ^1^ Biology Department Texas State University San Marcos Texas USA; ^2^ Institute for Molecular Life Sciences Texas State University San Marcos Texas USA

**Keywords:** chemical cue, *Gambusia affinis*, inhibitory control, microplastic, Poeciliidae

## Abstract

Urban stream syndrome is the collective term used to describe the physical and ecological degradation of streams draining urban lands that poses substantial threats to freshwater ecosystems. Among various consequences of urban expansion, microplastic pollution and shifts in predator–prey dynamics are prominent alterations to natural habitat that could impact the cognitive and behavioral responses of aquatic species. To explore how symptoms of urban stream syndrome impact the cognitive and behavioral responses of fish, we conducted two experiments using a delayed detour test to measure risk‐taking and inhibitory control in *Gambusia affinis*. In the first experiment, we hypothesized that *G. affinis* exposed to different concentrations of microplastics would show altered inhibitory control and risk‐taking. In the second experiment, we hypothesized that exposure to predator chemical cues during the detour task would alter inhibitory control and risk‐taking in *G. affinis*. We did not find significant differences in inhibitory control or risk‐taking in *G. affinis* exposed to microplastics or predator cues. We then compared the effect size and confidence intervals (CI) of these results with published results that used the same detour test to study inhibitory control and risk‐taking in *G. affinis* in response to different environmental conditions. Our investigations revealed that the CIs of the two studies presented here were larger than the CIs in the previously published studies. We consider potential changes to the experimental design that might have affected our ability to detect differences, such as the dimensions of the testing tanks. We also suggest extending the duration of the test to allow ample time for both exiting the starting chamber and solving the detour. We also propose considering the size and age of the species under study and adjusting the dimensions used in the detour paradigm design. Although our findings are specific to *G. affinis*, our results underscore the importance of considering aspects of the detour test design that are ecologically relevant to the study species when analysing cognitive and behavioral responses in fish. With our discussion, we contribute to the understanding of detour test methodologies and highlight potential ecological factors that could influence cognitive and behavioral outcomes.

## 1 INTRODUCTION

With expanding urbanization and subsequent increases in impervious cover, there is an increase in the volume of water runoff that enters streams draining urban areas. The increased flow of runoff can alter channel morphology, flow regimes, and patterns of sediment deposition in urban streams, reducing physical habitat complexity (Baruch et al., 2018; Booth et al., 2016; Vietz et al., 2016; Walsh et al., 2005). Runoff also carries plastic litter from urban areas that are then introduced into proximate streams, resulting in higher pollution loads in these habitats. Another symptom of urban stream syndrome is altered species composition, with population declines of sensitive species and increases in tolerant species. Changes in species composition could result in changes to predator–prey interactions in urban stream habitats. These significant alterations to the physical, chemical, and ecological properties in urban streams are known as “urban stream syndrome” (Walsh et al., 2005). The prevalence of urban stream syndrome suggests the necessity for species to respond and cope, possibly through alterations in cognition and behavior as a response to ecological degradation of urban streams.

Cognition includes learning, memory, and problem‐solving, and these abilities mediate the perception of environmental stimuli in the central nervous system (Shettleworth, 2001). Cognition is a mental process that is closely associated with behavioral outcomes, and the link is bidirectional (Blumberg & Wasserman, 1995; Shettleworth, 2001; Zentall, 2001). Inhibitory control is the outcome of the cognitive ability to regulate behavioral response and engage in alternative responses to achieve a specific goal or outcome (Kabadayi et al., 2018; Lucon‐Xiccato, 2024). Inhibitory control is a class of executive function that requires an animal to inhibit a predisposed response and engage in an alternative response that will lead to a more favorable outcome in the given context (Diamond, 2013). Given that behavior is frequently one of the initial traits to respond to change (Couzin & Heins, 2023), research in cognitive ecology can provide valuable insights into how altered environments affect cognitive abilities and behavior within individuals and across populations. One method broadly applied to study such ecological questions is a detour paradigm, which can be used to measure inhibitory control, learning, social learning, task switching, and route planning. The detour paradigm model presents an individual with a reward, but the direct path to reaching the reward is obstructed, creating a scenario in which the subject must use inhibitory control to take an active detour to acquire the reward. The detour paradigm can be applied to test many taxa across varied environments (Kabadayi et al., 2018; Lucon‐Xiccato et al., 2017; MacLean et al., 2014) and has been extensively used for teleost fish (reviewed by Lucon‐Xiccato, 2024).

Inhibitory control is likely to affect behavioral flexibility in animals when responding to unexpected environmental changes and in contexts important to organismal fitness, such as during foraging and in predator–prey interactions. For prey animals, inhibitory control is an important form of effective predator evasion (Evans et al., 2019); for example, juvenile chum salmon, *Oncorhynchus keta*, inhibit foraging behaviors when exposed to predation risk (Ryer & Olla, 1991). Indeed, predation pressure can prevent thorough investigation of surrounding habitat in fish (Beri et al., 2014). Inhibitory control is also important to spatial perception and could be elicited in stream environments when animals navigate around barriers such as rocks or vegetation to reach a goal such as returning to conspecifics in a shoal. However, pollutants in the environment, such as the increased microplastic loads associated with urban stream syndrome, could negatively affect cognitive abilities (Zala & Penn, 2004), such as inhibitory control, because they can cross the blood–brain barrier and affect fish behavior (Mattsson et al., 2017). Environmental influences on inhibitory control could also have associated changes in risk‐taking behavior in fish. When habitat is altered to the point in which the degradation threatens the survival of inhabitant populations, risk‐taking or exploratory behavior becomes critical to navigate to new environments and avoid stressors. In such situations, species need to first be willing to leave a refuge before they can explore a novel environment (Sloan Wilson et al., 1994). Inhibitory control and risk‐taking behavior play crucial roles in the overall fitness of fish, especially when they face rapid environmental changes in aquatic ecosystems. These cognitive and behavioral traits can be assessed using a detour paradigm.

The classic delayed detour design allows for measures of motivation and inhibitory control of a focal animal that must navigate an obstructed path to reach a reward. Motivation is a measure of an animal's willingness to reach a reward (Watts, 2001), and it is quantified in the detour test as the time the focal fish take to reach the plexiglass barrier that separates them from the reward. In our experiments, we utilized a plexiglass barrier to separate a focal fish from a social reward. Inhibitory control is then quantified as the time the individual takes to navigate around the barrier (out of sight of the reward fish) and reach the reward (solution speed). Measures from a delayed detour test can reflect an individual's perception and spatial awareness of the environment (Norman et al., 2019). Focal animals that solve faster in a detour test may have a better understanding of their environment's structure and the location of resources, potential threats, and available refuge. However, it is important to recognize that the physical design of the detour test could affect performance in the barrier task. Lucon‐Xiccato (2024) summarized some of the limitations of the detour design such as the distance to the goal and reward visibility for fish. For example, increased goal distance can make it easier to execute detours in primates (Diamond, 1990; Junghans et al., 2016) and fish (Gatto et al., 2018).

The objective of this article is twofold. First, to present our findings from two experiments that explore consequences of urban stream syndrome on inhibitory control and behavior using western mosquitofish, *Gambusia affinis. G. affinis* is a widespread species of live‐bearing fish that is of interest because populations successfully occupy a wide range of habitat types, including those that have been dramatically altered by urbanization. We investigated the impact of different amounts of microplastic exposure (aquatic pollutants) on inhibitory control and risk‐taking over time of repeated exposures. We hypothesized that varying degrees of microplastic exposure would be associated with differences in inhibitory control and risk‐taking behavior in *G affinis*. In our second experiment, we hypothesized that chemical cues of a predator may temporarily alter cognitive outcomes using a reaction norm approach. This is relevant to urban stream syndrome because another outcome is increased predation pressure due to less available refuge in areas of reduced habitat complexity.

Second, we compared the results from these two experiments to two published experiments using the same detour design to draw conclusions about how certain ecological and contextual factors could alter barrier task detour performance in fish. Finally, we measured repeatability of cognition and behavior in both experiments. Because some individuals may be consistently more efficient in inhibitory control, and this affects individual differences in cognitive performance, it is also useful to measure repeatability. Repeatability (*r*) is a measure that relates variation within individuals to the variation between individuals and indicates the consistency of the expression of a trait by individuals. Individuals need to be tested more than once to differentiate between variation in cognition and behavior due to variation existing among individuals and within individuals (Widemo et al., 1999). Repeatability reveals when the variation within individuals is less than between individuals and indicates the need to have larger sample size to detect response to treatments. Repeatability also indicates an upper bound estimate of heritability of a trait (Dohm, 2002; Lessells & Boag, 1987). Most relevant, though, is that fish may learn over time, so exploring repeatability provides insights into learning.

## 2 METHODS

### 2.1 Ethical approval

All protocols for this research were approved by Texas State University Animal Care and Use Committee IACUC #83. We declare no conflicts of interest.

### 2.2 Delayed detour test design

**FIGURE 1:**
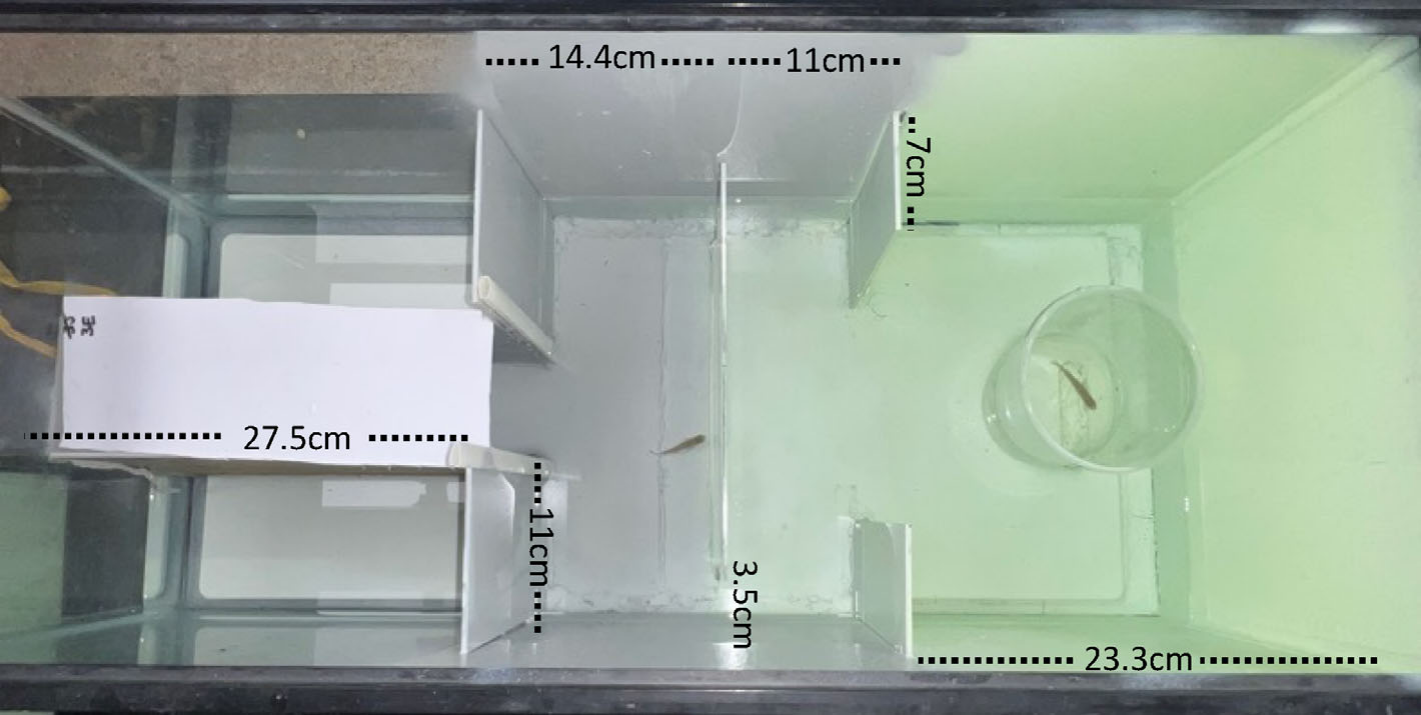
Dimensions of detour test used to test adult *Gambusia affinis*. Focal fish is placed in a covered starting chamber (left) and reward fish is placed in a 945‐mL transparent cup (right) positioned against the back aquarium wall. After a 10‐min acclimation period, the door to the starting chamber is lifted (not shown). (a) Exit time (s; risk‐taking) is measured by the time elapsed for the focal fish to leave the covered starting chamber. (b) Motivation (s) is measured by the time the fish takes to reach the plexiglass barrier (gray) after exiting the starting chamber. (c) Solving speed (s; inhibitory control) is measured by the time the focal fish takes to navigate through the gaps on either side of the plexiglass and reach the reward female.

To test cognitive performance in *G. affinis*, we used the same delayed detour design tank for all experiments outlined below. For all experiments we tested only female focal fish because this species is a live‐bearing fish, and we expect females to have larger costs to their behavior and cognition during the reproductive season. In our detour test design, we also use a female fish as a social reward because we were not interested in potential questions about mate choice. The delayed detour test design consists of using a visible goal in which the female social reward (enclosed in a 945‐mL transparent cup filled with 450 mL of water) is behind a clear plexiglass barrier and is visible from the focal fish's initial starting position (Figure 1). However, the reward fish is out of view (thus delaying gratification) when the focal fish moves toward the corridors it must pass through on either end of the barrier to reach the female reward. The plexiglass barrier presents a novel stimulus and therefore provides a useful method for comparing the ability to detour around a barrier to reach a reward across different populations and experimental scenarios. We altered the classic design by enclosing the starting chamber with opaque walls and a removable door following Guzman et al. (2023) that was modified from Wallace et al. (2020). The inclusion of an enclosed starting chamber allows for an additional behavioral measure of risk‐taking. After acclimating the focal fish in the enclosed starting chamber for 10 min, the door is lifted, and the time elapsed for the fish to exit the starting chamber and move into the bright, open arena can be used as a measure of risk‐taking (White et al., 2013). After exiting the boundary of the starting chamber, the time the focal fish takes to reach the plexiglass barrier (obstructing the direct path to the visible social reward) provides a measure of individual motivation. The time to reach the reward female after reaching the barrier was recorded as solving speed. The tank was painted grayish blue for the starting zone and then green for the solution zone. Studies of scototaxis have found that four commonly used fish species (including guppies, *Poecilia reticulata*) for neuroethology show preference for darker areas and avoid white areas (Maximino et al., 2010), so we avoided the use of white background. The tank was lit from above with 40 W 6500 K white LED lights but offset so as not to cause shadows or reflections on the tank. The tank was filled with 6 cm of dechlorinated water at room temperature (20°C), and water was replaced daily. All behavior was recorded using a high‐resolution webcam (1080 p with 30 fps) placed vertically above the detour test aquariums, and data were obtained from watching the video recordings. A focal fish was placed in the covered starting chamber and given a 600‐s acclimation period. After acclimation, the door to the starting chamber was raised, and fish were given 600 s to exit and complete the detour. Subsequently we measured time to exit for risk‐taking, time to reach the barrier as motivation, and time to reach the reward as a measure of inhibitory control from the video recordings. A single observer scored all recordings.

### 2.3 Microplastics

We investigated the effects of microplastic (polystyrene) exposure on the inhibitory control and risk‐taking behavior in *G. affinis* using the delayed detour test, described previously, in an experiment that lasted 33 days. We collected adult *G. affinis* in Hunt, Texas, on July 27, 2021. Fish were maintained in 38‐L tanks with rocks, plants, and a filter until testing started 18 days later. Fish were maintained at 26°C and fed Tetra flakes ad libitum daily. Fish (*n* = 15) were tested in three treatments of varying microplastic exposure: high microplastic (*n* = 15) concentrations (282 μL: H), low microplastic (*n* = 14) concentrations (9 μL; L), and no (*n* = 15) known microplastics (C). Each fish was housed independently in transparent holding containers (945 mL) with holes within a 38‐L aquarium to facilitate a continuous exchange of visual and olfactory cues among conspecifics as this species shoals (Pyke, 2005). One or more fish died in each treatment before the experiment ended. We purchased microplastics that consisted of 10‐μm polystyrene blue, fluorescent spheres (ThermoFisher, F8829). Following Boyero et al., (2020) for the high‐exposure treatment, we pipetted different levels of microplastics, starting at 3.6 × 10^−6^ part. mL^−1^, into each container of fish with brine shrimp to increase the chance of microplastic ingestion. *G. affinis* were exposed to two independent pulses of environmentally relevant levels of microplastic to mimic exposure after rainfall that occurs in natural conditions: once on day 1 and again on day 12. After each exposure to microplastics, one person (KI) tested fish from each treatment in the delayed detour test (explained above) on days 2 and 13.

### 2.4 Predator cue

We used 60 female *G. affinis* on July 27, 2023, and 3 bluegill sunfish, *Lepomis macrochirus*, on August 2, 2023, and transported them to the laboratory. The female mosquitofish were maintained in 38‐L tanks with rocks, plants, and a filter until testing started August 6–26, 2023. We maintained the sunfish in the laboratory for 5 days in a 75‐L tank and fed them live earthworms for a neutral diet. Five days were allowed for the excretion of any prior dietary cues and the accumulation of neutral diet cues. After this, all three bluegill sunfish were housed for 24 h in clean (3% hydrogen peroxide) unfiltered tanks. We determined the volume of each bluegill sunfish by displacement and used 230 mL of water per 1 mL of water displaced. The water from the aquarium was mixed and then frozen at −20°C in 50‐mL aliquots for use as sunfish predator chemical cue. To examine behavioral and cognitive response of *G. affinis* to the chemical cues of a sunfish predator, we used the same delayed detour test as described previously. Once placed in the detour test, female *G. affinis* were exposed to sunfish chemical cue first (*n* = 32) or to water control cue first (*n* = 28) in a reaction norm design. The next day, the fish were exposed to the opposite cue (water control or predator cue). During the 600‐s acclimation period for each the focal fish, we introduced water from the defrosted sunfish chemical cues or water (control) cues into the far back left of the reward section of the detour aquarium. We injected 50 mL of the predator cue at a rate of 1 mL/s using a syringe and tubing positioned a centimeter below the water line to decrease disturbance. We injected the cue ahead of releasing the focal fish so the cues had time to disperse toward the focal fish. After the 600‐s acclimation period (and cue introduction), the door to the starting chamber was lifted, and the focal fish was given 600 s to solve the detour test. The two treatments were performed on sequential days with 10 fish tested a day from August 8 to 26, 2023. The reaction norm design allows for the analysis of a single individual across two distinct treatments, enabling a comparison of an individual behavior against its own baseline and statistically controlling for individual variation. A single observer (G.H.) recorded behavior and cognition from a video recording of each trial.

### 2.5 Statistical analyses

To analyse the relationship between treatments in the two experiments, we used a Cox's proportional hazards model (Coxme R package) with censored data to account for fish that did not exit or exited but did not solve. We used separate models for exit time, motivation, and solution speed. In all models we included the standard length (in millimeters) and treatment as a factor and ID as a random effect. We present non‐standardized effect sizes with 95% CI based on false discovery rate (FDR)‐adjusted values. We used the R package rtpR in R to calculate an adjusted repeatability (r) with a linear mixed model (LMM)‐based approach using the restricted maximum likelihood (REML) method (Dingemanse & Dochtermann, 2013; Nakagawa & Schielzeth, 2010) for the two experiments presented in this article. We then compared the results from the microplastic exposure study and the predator cue study to two published studies that used the same detour design tanks. The first of the previously published studies we used for comparison was designed to explore the cognitive and behavioral performance of populations *G. affinis* from varying degrees of environmental complexity. We defined habitat complexity as the degree variation in physical habitat structure (sediment and vegetation), with high variation equating to higher complexity. For this experiment, we collected female *G. affinis* from nine populations (*n* = 40 per population) that persisted in different levels of habitat complexity that we assigned as low, medium, or high complexity (Irwin et al., 2024). In the other previously published study, we tested the cognitive performance of female *G. affinis* (*n* = 18) when the fish were maintained and tested at 21°C versus 31°C (Guzman et al., 2023). We then used a forest plot to compare the effect sizes and 95% CIs across boldness, motivation, and solution speed with the treatments of the microplastic and predator cue experiments with the results of our two published articles to explore the variance in fish across these experiments.

## 3 RESULTS

### 3.1 Microplastics

**FIGURE 2:**
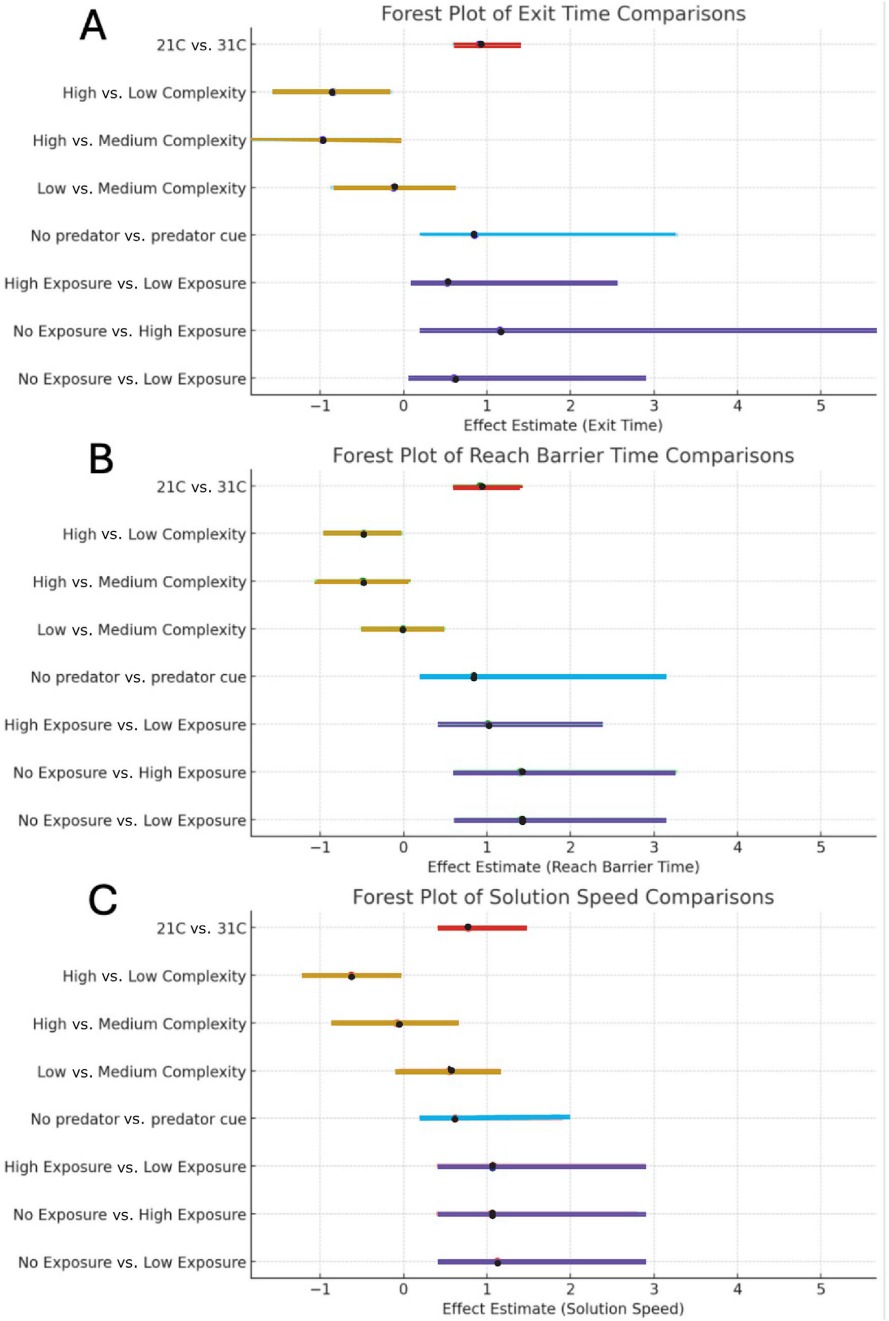
Forest plot comparing the non‐standardized effect sizes with 95% CI based on false discovery rate (FDR)‐adjusted values for (a) exit time (s), (b) reach barrier (s), and (c) solution speed (s) across four experiments (labeled with different colors: temperature differences [*n* = 18]; habitat complexity experiment [*n* = 40]; predator cue experiment [*n* = 32]; and microplastic exposure [*n* = 15]). Larger effect sizes indicate substantial differences in response time to exit time, reach barrier, or solution speed. Negative estimated effect size indicates shorter response time, and positive estimated effect size indicates longer response time. CIs excluding 1 are significant.

In our microplastic experiment, we divided *G. affinis* into three treatments and exposed them to varying concentrations of microplastics in their holding aquariums: control (no plastics), low microplastics, and high microplastics. Then, we measured their boldness and inhibitory control on two different testing periods (days 1 and 2) using a detour test. For the control treatment, 4 of 13 total fish exited the starting chamber and solved the detour test on test day 2 (Table 1). For the low microplastic exposure treatment, 7 of 14 total fish exited the starting chamber and solved the detour test on test day 2 (Table 1). For the high microplastic exposure treatment, 4 of 14 total fish exited the starting chamber and solved the detour test on test day 2 (Table 1). With few fish exiting and fewer solving, it is not surprising that we found no significant difference in exit time (s) when comparing low exposure treatment to control (Figure 2). We did not find a significant difference in exit time (s), time to reach barrier times (s), or solution speed across any treatments (Figure 2). However, when we considered all treatments together, fish exit time (s) was repeatable (*r* = 0.51 ± 0.122; 95% CI = 0.223, 0.694; *p* = 0.0003) and motivation (s) was repeatable (*r* = 0.916 ± 0.032; 95% CI = 0.834, 0.959; *p* < 0.0001), but solving speed (s) was not repeatable (*r* = 0.0 ± 0.119; 95% CI = 0, 0.401; *p* = 0.5). Note that 26 fish were removed due to not solving.

**TABLE 1:** Proportion of female *Gambusia affinis* that exited the starting chamber, those that exited but did not solve, and those that exited and solved the detour test across three microplastic exposure treatments and two testing days.

Treatment	Test day	Exited	Exited but did not solve	Exited and solved
Control	1	12/15	3/15	9/15
	2	8/13	4/13	4/13
Low microplastic	1	13/15	6/15	7/15
	2	11/14	4/14	7/14
High microplastic	1	11/15	2/15	9/15
	2	8/14	4/14	4/14

### 3.2 Predator cue

We found that 4 of 32 fish did not exit the starting chamber when exposed to control water cue first, and 2 of 28 fish did not exit when exposed to predator chemical cues first. Of those that exited, 15 fish did not reach the reward fish (solve) in both treatments. We did not find a significant difference in exit time (s), motivation (s), or solving speed (s) between predator and no predator cue treatment exposures (Figure 2). We found that time to exit was repeatable (*r* = 0.842 ± 0.061; 95% CI = 0.7, 0.923; *p* < 0.0001), but time to reach barrier (*r* = 0.0 ± 0.092; 95% CI = 0.0, 0.299; *p* = 1) and solving speed (*r* = 0.022 ± 0.108; 95% CI = 0.0, 0.367; *p* = 0.456) was not repeatable.

### 3.3 Effect size and variance

The forest plots indicate that the habitat complexity and temperature difference experiments have the narrowest CIs, indicating that the results are more precise. But only the habitat complexity experiment showed significant response to the treatments. This experiment had the largest sample size, which provides a more reliable estimate of the true effect of the treatments, whereas the experiments with a smaller sample size may indicate an overestimate of the true effect or random variation. Note that the temperature experiment, despite having smaller samples sizes, had more precision than the other two studies with smaller sample sizes.

## 4 DISCUSSION

Urban expansion and increases in impervious cover result in more water runoff that enters water bodies draining urban areas. This runoff carries microplastics that enter urban streams, changes flow patterns that can lead to habitat degradation, and alters predator–prey relationships. Resulting microplastic pollution and changes in predator–prey interactions could affect cognitive and behavioral responses in *G. affinis*. Understanding how tolerant fish respond to changes in their environment is critical to future conservation efforts. Using a delayed detour test, we analysed risk‐taking and inhibitory control in the context of varying environmental conditions to unravel how tolerant species respond to human‐induced environmental changes.

### 4.1 Microplastics

We did not find a significant effect of microplastic exposure on risk‐taking behavior or inhibitory control of *G. affinis*. The fish were tested on two separate days, and although we would like to investigate a correlation for solving speed between days 1 and 2 to see if the fish were learning, we could not conduct a precise analysis given our small sample size. Additionally, fish that did not solve the test were given a score of 600 s, and this weights the results to have longer solving times, especially for testing day 2, considering that all fish on testing day 1 had to solve. From an ecological perspective, we could not reject the hypothesis that microplastics affect cognition. Another consideration is that *G. affinis* had variable levels of microplastic ingestion regardless of exposure level thus limiting differences observed between treatments. Microplastics are known to enter the body passively through the gills (Kashiwada, 2006; Lu et al., 2016), but this is dependent on the plastic size. We followed the methods of another experiment that successfully incorporated the plastics into tadpoles, so we think this was not likely the case. Despite evidence that nanoplastics can penetrate the blood–brain barrier (Mattsson et al., 2017), we do not see evidence of an effect. Mattsson et al. (2017) found that the effects of nanoplastics on exploration behavior were not observed until 2 months after exposure. We only tested *G. affinis* within 1 month of microplastic exposure, so this may also account for the lack of change in inhibitory control.

### 4.2 Predator cue

We did not find a significant difference in inhibitory control or risk‐taking in *G. affinis* whether or not they were exposed to the diet cues of predatory sunfish. This was unexpected because fish from populations that are exposed to more predators make more errors in maze solving and do not explore their habitat as well (Beri et al., 2014). One explanation may be that we extracted the chemical cues from small sunfish predators, and so their secretions might not have been perceived as a threat to the focal *G. affinis*.

In the predator cue experiment, we used a reaction norm design, leveraging the capacity to analyse a single individual across the two distinct treatments. This approach reduces the number of fish needed by subjecting each individual to both treatments, allowing for an analysis of their behavior against themselves so that any individual variation is controlled statistically. The fact that the CI was narrower for most of these measurements also supports the hypothesis that using a reaction norm design can increase the chance of observing a significant effect with smaller sample sizes, if you can get the fish to respond better to an experimental design that improves chances of exiting. Nonetheless we did not find any significant differences across the predator cue versus no predator cue treatment, and the CI range was large, indicating a lack of precision in the results. An alternative hypothesis is that we used only diet chemical cue from the predator instead of alarm cues from a predator‐eating conspecifics (Schreckstoff), which elicit stronger antipredator responses (Chivers et al., 1996). We suggest both increasing samples size and using Schreckstoff in a future experiment asking similar questions about the effects of predator cues on the cognition and behavior of focal individuals.

### 4.3 Detour design methods

The design of the delayed detour test and the time allotted for focal fish to solve the detour may need to be reevaluated. Our delayed detour design followed that of Wallace et al. (2020), and they did not detect significant differences when comparing cognitive performances between sexes of *G. affinis*. But our design added an additional step to measure exit time. By adding the behavioral component of exit time from a starting chamber into our detour test design, we also introduce the possibility that some fish will not exit the starting chamber and therefore would not be considered in an analysis for exit time or solving speed, reducing our total sample size. Initially, we did not anticipate that a considerable number of individuals would not exit, as most fish exited the chamber in Guzman et al., (2023). Upon comparing the effect sizes and CIs between the two studies presented here (the microplastic and predator cue experiment) and two published studies (the environmental complexity and temperature difference experiments), we observed greater CIs in the microplastics and predator cue experiments compared to our two previously published articles. This comparison emphasizes the importance of considering the CIs when interpreting results. Our two experiments presented here had large CIs indicating lower precision. It may be that the results from the predator cue experiment had lower CI (in two of the three measures) because we tested the same individuals across the two treatments (reaction norm). The results from the temperature difference experiment for exit time and reach barrier had the lowest CI indicating that the precision in this experiment was higher, and the consistency across the two behavioral measures strengthens the reliability. In our temperature difference experiment, fish were given more time to solve the detour test (10 min to exit, and 10 min to solve the detour), which could account for the narrower CI. Larger sample sizes (*n* = 40 vs. 20) used in the environmental complexity experiment yielded results that were more precise and reliable. However, this does not entirely account for the low precision in the predator cue experiment. An alternative hypothesis is changing the length of exposure to each treatment before testing the fish in the detour design. In the complexity experiment, fish were exposed to varying conditions for their entire lifetime, whereas in the temperature experiment, fish were exposed to the treatment for 30 days. In contrast, fish in the microplastic experiment were exposed to the treatment for only 18 days, and fish in the predator cue experiment were exposed to cues only during the detour test. These differences in exposure time may have contributed to the varying levels of precision observed across experiments, suggesting that both sample size and duration of treatment exposure are important factors in determining the reliability and precision of experimental outcomes.

Repeatability reveals when the variation within individuals is less than between individuals. When we examined repeatability, we found that exit time (s) was repeatable in both the microplastic and predator cue experiments, indicating consistent difference between individuals, but not so for solving speed. It is important to note that the predator cue experiment has consistent responses, and this could reflect high repeatability and not learning. The lack of repeatability for solving speed (inhibitory control) may be an outcome of fewer individuals solving the experiment, which is also affected by the number of individuals that exited. We suggest that adding time for the fish to exit could diminish the issue of low repeatability and high overall variance we found. Guzman et al. (2023) gave fish 600 s to leave the starting chamber, but they had more fish solving the experiment and high repeatability.

In general, experiments conducted in captivity could generate variation in behavioral and cognitive performances given differences in individual responses to captive conditions. Given that individual perception of the testing environment could differ based on prior experience in captivity (Kluen et al., 2022), another important design element to consider is the duration fish were kept in the laboratory prior to the beginning of experimentation. For the microplastic experiment, wild fish were caught in the field and brought back to the laboratory for a 48‐h acclimation period to captive conditions in the laboratory before experiments began. For the predator cue experiment, focal fish had been maintained in the laboratory for months prior to experimentation. This previous experience in captivity could have introduced a carryover effect and impacted the time to acclimate to the novel testing environment and influence responses to novel stimuli (Kluen et al., 2022). Butler et al. (2006) found that wild birds that spent longer in captivity were less successful in completing behavioral trials compared to individuals with shorter acclimation periods. When we compared behavior and cognition scores between testing days 1 and 2 of the microplastic experiment, we found a marginally significant effect of testing day on exit times, with fish taking longer to exit on day 2 regardless of treatment. Although insignificant, there was also a trend toward faster solution speeds on day 2.

Here we suggest additional alterations to the delayed detour test that can be used to in future experiments in freshwater fish. In all our experiments *G. affinis* were provided 600 s in total to exit the starting chamber and complete the detour. Some individuals did not leave the starting chamber or were much later to leave and did not solve the detour in 600 s and could not be scored for inhibitory control. To accommodate for such differences in exit time in future experiments, we found that giving individuals 600 s to exit the chamber and then 600 s to solve the detour the moment after exiting the starting chamber gave fish more opportunity to reach the reward, and made measures of exit time and solving speed independent. The results of our experiments are specific to *G. affinis*, but performance outcomes in detour test tanks vary across populations and species (Santacà et al., 2019, Irwin et al., 2024). Santacà et al. (2019) found that zebrafish, *Danio rerio*, were successful solvers when they could rely on chemical cues. Our detour tests did not include chemical cues from the reward fish (reward fish were contained in a transparent cup), so the addition of chemical cues could be useful to increase the motivation of these fish to respond. However, we did not drain the water out of the experimental tanks before each trial, and therefore, chemical cues from previously tested focal fish were present in successive trials. This might have influenced focal fish exit time and participation in the detour, but Guzman et al. (2023) did do water changes before the beginning of each trial and did not find a significant effect of treatment on the cognitive performance of *G. affinis*.

**FIGURE 3:**
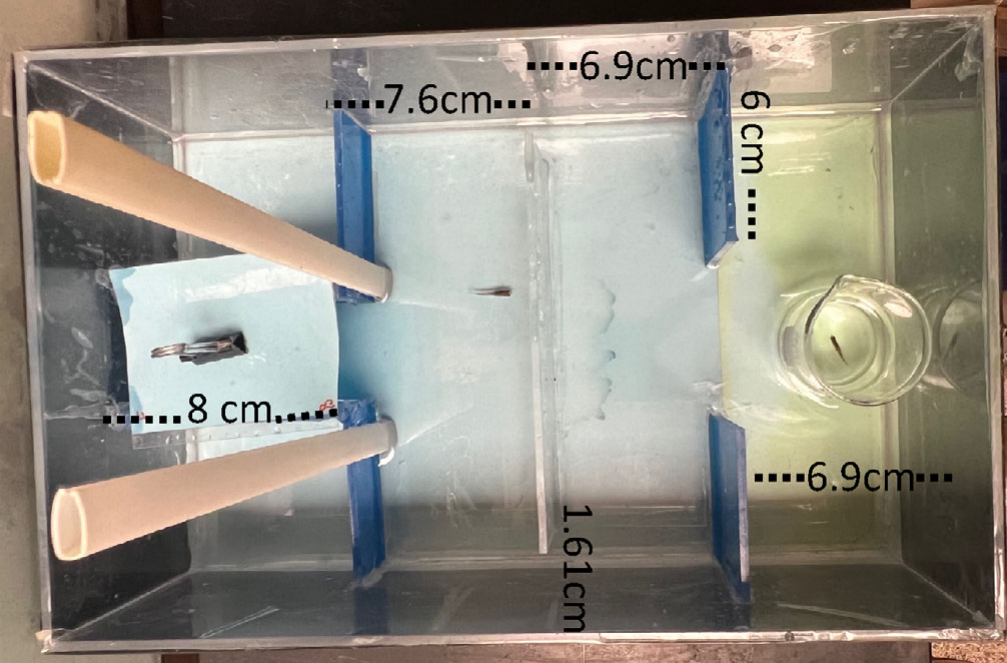
Photograph and dimensions of detour test used for juvenile fish; there is a 1.6‐cm gap that the fish can swim around the barrier.

Another factor to consider when explaining variation in performance outcomes is the physical design and dimensions of the experimental tank. The physical size of the experimental tank has been shown to affect fish performance success in cognition assays (Jones et al., 2023), and so it is important to consider an ecologically relevant amount of space a species might move through, and how the scale of these distances differs throughout its life. For example, when juvenile fish are tested, it is important to recognize that they are not able to swim as far as adults and may be found in shallower habitat. In another study from our laboratory (Saldivar et al. in prep.), we tested the cognition of juvenile *Heterandria formosa* another species of live‐bearing fish, and our findings suggest that juvenile *H. formosa* were more likely to exit when the starting chamber was shorter in a tank about 39% smaller than the tank we used for adults (Figure 3 vs. Figure 1). Additionally, we designed the distance from the threshold of the starting chamber to the barrier relatively longer (52% of the larger tank) than in the tank we used for the adult fish (Figure 1). This adjustment aimed to introduce more variance in the time taken to reach the barrier, thereby increasing the likelihood of detecting significant differences between treatments. We also designed the distance to the reward to be shorter (9.3 cm vs. 26.3 cm or 35% less), which may have limited the success of fish from solving, as Gatto et al. (2018) found that fish are more likely to solve a detour task if the reward is further from the barrier. However, in a different type of cognition assay design, Jones et al. (2023) found that larger T‐mazes with longer arms resulted in longer latencies to leave the exit chamber and fewer successful participants in the trial. In our larger detour tank design (Figure 1), the distance to the reward was 26.3 cm, which was slightly longer relative to the total size of the testing chamber. In our microplastic experiment, 34% of the fish reached the reward, but in the predator experiment, 62% did not solve the first time they were tested. Thus, there are differences in solving rates that might differ based on multiple variables—all of which we have yet to determine.

Urban stream syndrome, driven by urban expansion and increased impervious cover, poses significant challenges to aquatic ecosystems. Resulting microplastic pollution and changes to predator–prey interactions loads could affect cognitive and behavioral responses in *G. affinis*. By analysing risk‐taking and inhibitory control in this species, we aimed to unravel behavioral and cognitive responses to varying environmental conditions. Despite efforts to investigate the effects of microplastics exposure and predator cues, our study encountered limitations, primarily due to sample size constraints. Although our findings did not reveal significant differences in cognition and behavior, we identified the importance of sample size for a more precise analysis of detour test scores. In addition to increasing sample size to better capture individual variation, future experiments should adjust the detour test protocol to extend the allotted time—10 min for the focal fish to exit and 10 min for the focal fish to solve the detour. Overall, our experiments helped identify design elements of the detour test and ecological factors that could affect measures of risk‐taking and inhibitory control from detour test experiments.

## AUTHOR CONTRIBUTIONS

K.I. contributed to: conceptualization of manuscript idea, conceptualization of research topic, funding acquisition, investigation, methodology, data curation, data analysis, data visualization and writing ‐ original draft, review, and editing. G.H. contributed to: conceptualization of research topic, investigation, and data curation. C.R.G. contributed to the development of the manuscript idea, conceptualization of research topics, funding acuisition, data analyses, data visualization and writing ‐ original draft, review, and editing.

## FUNDING INFORMATION

This research was partially funded by the Texas State University Masters Fellowship, however, there is not a grant number for this.

## CONFLICT OF INTEREST STATEMENT

The authors declare no conflicts of interest.

## Data Availability

Data and R code are available from Figshare.
